# Case report: Mounier-Kuhn syndrome

**DOI:** 10.4103/0971-3026.40955

**Published:** 2008-11

**Authors:** Satish Kachhawa, ML Meena, Gaurav Jindal, Bharat Jain

**Affiliations:** Department of Radiodiagnosis, Sardar Patel Medical College, Bikaner, India

**Keywords:** Mounier-Kuhn syndrome, tracheobronchomegaly

## Abstract

Tracheobronchomegaly or Mounier-Kuhn syndrome is a rare disorder characterized by marked dilatation of the trachea and main bronchi, bronchiectasis, and recurrent respiratory tract infections. The etiology of this disorder is uncertain and the clinical presentation is variable. The diagnosis is usually made on the basis of the characteristic CT scan findings. We report a case in a 21-year-old man presenting with recurrent lower respiratory tract infections.

Mounier-Kuhn syndrome or tracheobronchomegaly is a rare clinical and radiological entity characterized by marked dilatation of the trachea and bronchi and recurrent lower respiratory tract infections. Diagnosis is usually made on CT scan. The condition is known by a number of different names, e.g., trachiectasis, tracheobronchopathia malacia, tracheomegaly, and multiple tracheal diverticula.

## Case Report

A 21-year-old man was admitted to our institute with complaints of recurrent lower respiratory tract infections since childhood, presenting as episodes of productive cough with fever. He was asymptomatic in between these episodes. The patient was a nonsmoker and there was no family history of a similar illness. The chest radiograph showed enlargement of the trachea and bronchi and bilateral bronchiectasis. 

**Figure 1:**
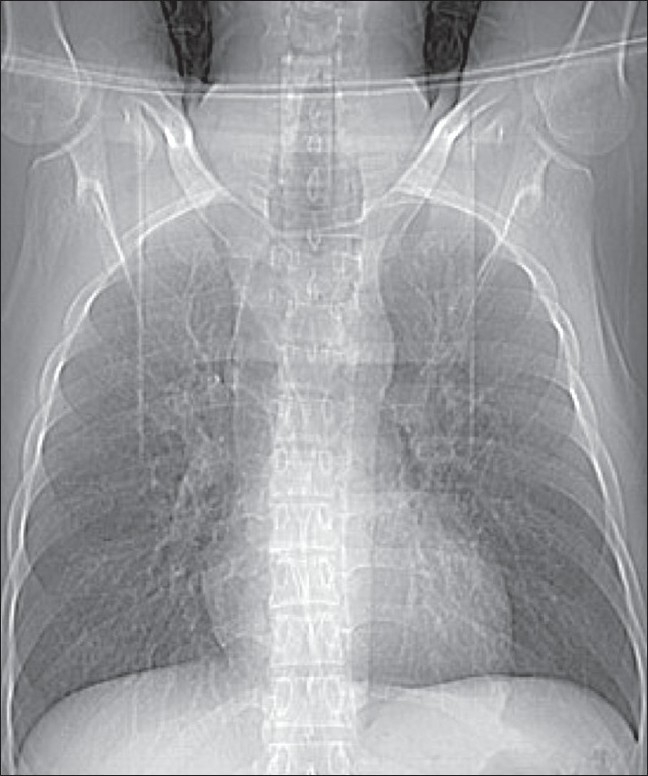
CT scannogram shows enlargement of the trachea and bronchi and bilateral bronchiectasis

**Figure 2 (A–D):**
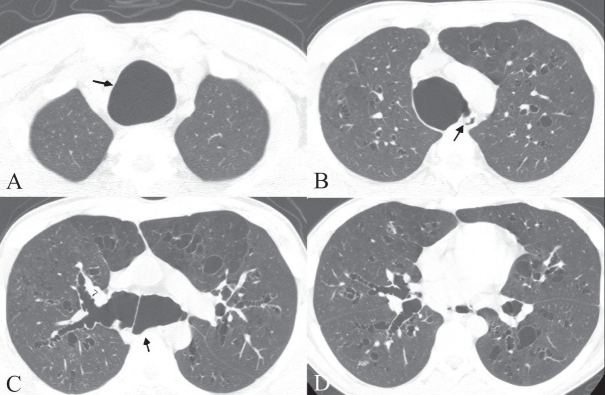
Axial high-resolution CT images show marked dilatation of the trachea (arrow in A), posterior tracheal diverticula (arrow in B), dilatation of the main bronchi (arrows in C), multiple diverticula (arrowheads in C), and bilateral cystic bronchiectasis (D)

CT scan of the chest was performed. The scannogram [Figure 1] showed tracheobronchomegaly. The trachea was grossly dilated, with a diameter of 4.1 cm [Figures 2A and B], while the right and left main bronchi had diameters of 2.6 and 2.9 cm [Figure 2C], respectively. Multiple diverticula and areas of scalloping were seen between the cartilaginous rings in the trachea and right and left main bronchi [Figures 2A and B]. Cystic bronchiectasis was seen in the lung parenchyma bilaterally [Figure 2D]. 

Fiberoptic bronchoscopy revealed a dilated trachea with prominent tracheal rings and widening of the bronchial tree bilaterally; mucopurulent secretion was seen at places.

## Discussion

Congenital tracheobronchomegaly, or the Mounier-Kuhn syndrome, is a rare clinical and radiological entity described by Mounier and Kuhn for the first time in 1932.[1] The syndrome is characterized by marked tracheobronchial dilatation.[1–8] Most cases present in the third or later decades with recurrent respiratory tract infections.[6] Although the etiology is uncertain, it is believed to be due to the lack of smooth muscle and elastic connective tissue in the trachea and main bronchi, leading to sacculations and the formation of diverticula between the cartilaginous rings.[9,10]

Disorders such as sarcoidosis, usual interstitial pneumonia, and cystic fibrosis, which cause severe fibrosis of the upper lobes, may also exert sufficient tracheal traction to result in tracheal enlargement.[9] Certain other conditions such as Marfan syndrome, Ehlers-Danlos syndrome, Kenny-Caffey syndrome, ataxia-telangiectasia, connective tissue diseases, Brachmann-de Lange syndrome, Bruton-type agammaglobulinemia, ankylosing spondylitis, cutis laxa, and light chain deposition disease are also associated with secondary tracheobronchial enlargement.[11] Most cases, however, are sporadic and show no evidence of associated connective tissue disease,[2,11] as was the case in our patient also.

On CT scan, the diagnosis is made when the transverse diameter of the trachea measures greater than 3 cm and that of the right and left main bronchi exceeds 2.4 cm and 2.3 cm, respectively.[7] The diameters in the present case were 4.1, 2.6, and 2.9 cm, respectively. Apart from the tracheobronchial enlargement, diverticula are also seen between the cartilaginous rings. Recurrent episodes of pneumonia usually lead to bronchiectasis, as was seen in our case.

As tracheobronchomegaly can be overlooked on plain films, patients who have chronic respiratory infections should have a CT scan done in to rule out underlying predisposing conditions such as this.
